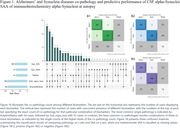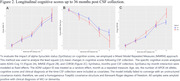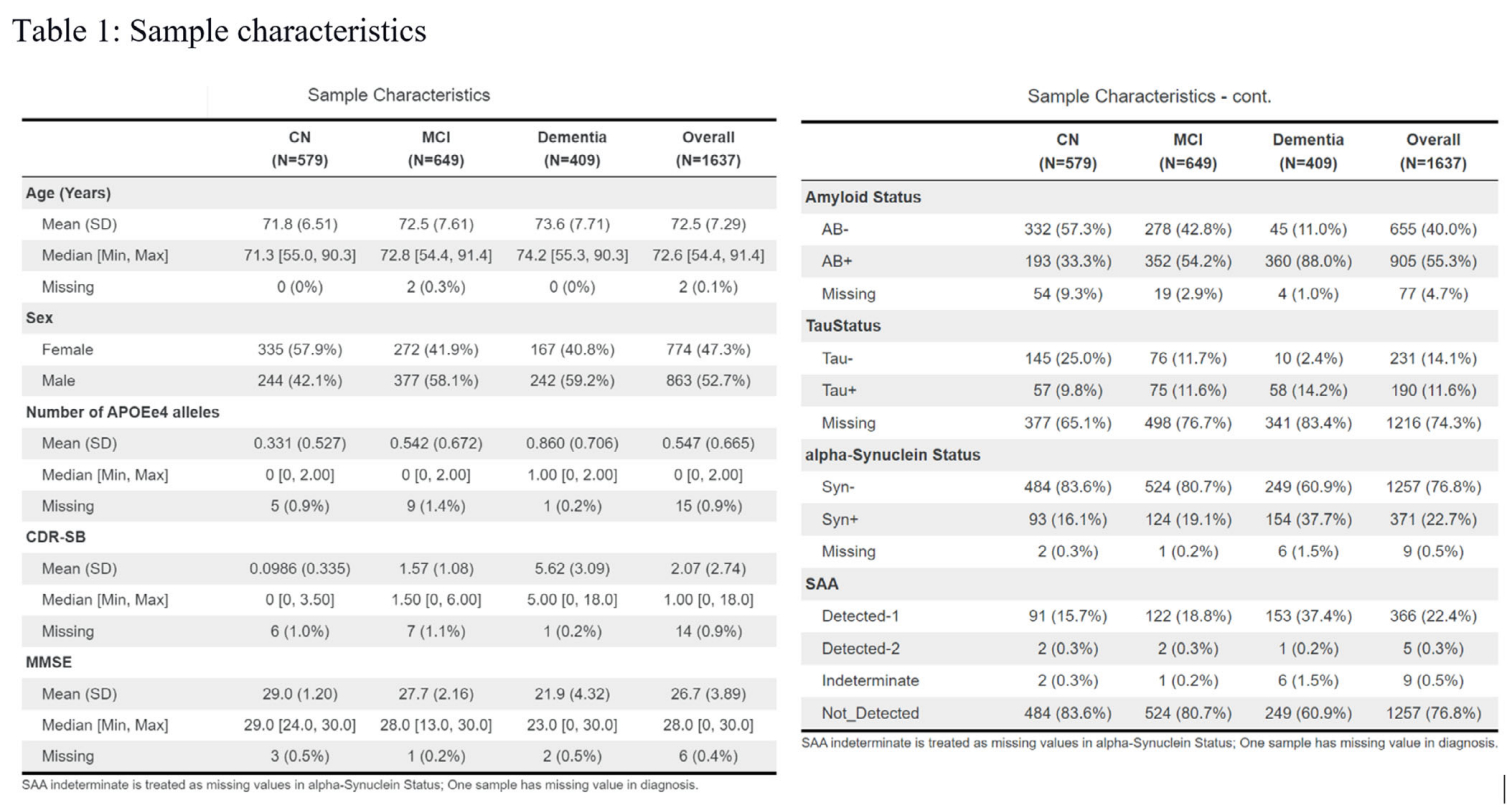# Cognitive changes in co‐pathological Alzheimer’s disease and Synuclein diseases: A study of amyloid, tau, and alpha‐Synuclein biomarkers in the ADNI dataset

**DOI:** 10.1002/alz.091662

**Published:** 2025-01-09

**Authors:** Jian Wang, Ming Lu, Sergey Shcherbinin, Amanda Morris, Saima Rathore, Leonardo Iaccarino, Michael Pontecorvo, Emily C. Collins, Mark Mintun

**Affiliations:** ^1^ Eli Lilly and Company, Indianapolis, IN USA; ^2^ Eli Lilly and Company, Philadelphia, PA USA

## Abstract

**Background:**

Alzheimer’s and Synuclein diseases are characterized by distinct biomarkers and frequently co‐occur, suggesting potential interactions between their pathological pathways. This study leverages amyloid and tau PET imaging, along with CSF Phosphorylated tau (P‐tau) and alpha‐synuclein measurements from the Alzheimer’s Disease Neuroimaging Initiative (ADNI) to investigate the impact of co‐pathology on cognitive functions.

**Method:**

We conducted an analysis using ADNI data (Table 1) from the 2024‐01‐08 download, including results from the CSF alpha‐Synuclein Seed Amplification Assay (SAA, 2023‐09‐29 release, 1637 samples out of 1638 records included in the analysis). The diagnostic performance of the CSF alpha‐Synuclein SAA was assessed by comparing it to autopsy alpha‐Synuclein immunohistochemistry (IHC) findings. Cognitive and functional assessments were examined in relation to amyloid, tau, and alpha‐synuclein biomarkers, both individually and collectively. Mixed‐model repeated measures (MMRM) analysis was utilized to evaluate ADAS‐Cog13, MMSE, and CDR‐SB score change up to 36 months following CSF collection, adjusting for age, sex, number of APOE e4 alleles, cognitive scores and clinical diagnoses at CSF collection.

**Result:**

The CSF alpha‐Synuclein SAA showed a strong agreement with postmortem alpha‐Synuclein IHC (Figure 1). The probability of positive alpha‐Synuclein (Syn+) was associated with aging and the severity of clinical diagnosis (cognitively normal to mild cognitive impairment to dementia). Cross‐sectionally, subjects with Amyloid+Syn+ had worse cognitive scores compared to those with Amyloid+Syn‐ status. Investigation of the relationship between alpha‐Synuclein and tau biomarkers was pending on additional samples in upcoming data release. MMRM analysis (Figure 2) revealed that longitudinally subjects of MCI or dementia with Amyloid+Syn+ status declined faster in ADAS13, MMSE, and CDRSB scores than those with Amyloid+Syn‐ status. These differences were statistically significant at 36 months post‐CSF collection for MMSE (p < 0.01) and CDR‐SB (p < 0.05).

**Conclusion:**

The worse cross‐sectional cognitive scores, as well as faster longitudinal decline in Amyloid+Syn+ vs Amyloid+Syn‐ subjects suggest that the co‐occurrence of these biomarkers indicates a more aggressive trajectory of dementia. These observations reinforce the importance of comprehensive biomarker profiling in predicting cognitive decline and underscore the need for further studies to investigate the complex dynamics of co‐pathology in Alzheimer’s and Parkinson’s diseases.